# The Impact of Job Strain on Occupational Access to Firearms and Firearm‐Related Suicide Among US Workers

**DOI:** 10.1002/ajim.70078

**Published:** 2026-04-01

**Authors:** Victor A. Soupene, Jonathan Davis, Joseph E. Cavanaugh, Jonathan M. Platt, Paul A. Romitti, Carri Casteel

**Affiliations:** ^1^ Department of Occupational and Environmental Health, College of Public Health University of Iowa Iowa City Iowa USA; ^2^ Department of Emergency Medicine, Carver College of Medicine University of Iowa Iowa City Iowa USA; ^3^ Department of Biostatistics, College of Public Health University of Iowa Iowa City Iowa USA; ^4^ Department of Epidemiology, College of Public Health University of Iowa Iowa City Iowa USA

**Keywords:** access to lethal means, firearms, occupation, stress, suicide

## Abstract

**Introduction:**

Firearm‐related suicide rates are notably high among workers such as police officers and farmers. One risk factor is occupational access to firearms, but other occupational characteristics, such as job strain, are less understood. This study examined the impact of job strain on the association between occupational access to firearms and firearm‐related suicide.

**Methods:**

This cross‐sectional study used National Violent Death Reporting System data for 2013–2019 (*n* = 81,196). The outcome was firearm‐related suicides, which were compared to suicides by other lethal means. The exposures included workers who used firearms as part of their job. Job strain was measured through the combination of job demand and job control measures. Job strain served as an effect modifier of the association between occupational access to firearms and firearm‐related suicide. Adjusted odds ratios with 95% confidence intervals were estimated using multivariable logistic regression models, with stratified analyses for biological sex.

**Results:**

Working in a high‐strain job (characterized by high job demands and low job control) significantly increased the odds of firearm‐related suicide for both male (OR = 2.4, 95% CI: 1.9, 3.0) and female (OR = 4.0, 95% CI: 2.4, 6.5) decedents, compared to those with no occupational access to firearms and working in low‐strain jobs (characterized by low job demands and high job control).

**Discussion:**

Reducing job strain in occupations with access to firearms may help decrease firearm‐related suicides. Future research should explore the role of social support and additional individual‐level factors, including access to personal firearms and the temporality of psychosocial factors related to occupation.

## 1 Introduction

Suicide is a growing problem among US workers. Firearm‐related suicides contributed to more than 10% of all fatal injuries in the United States (US) during 2001–2020 [1]. Among all 23 high‐income nations, the US has the highest firearm‐related suicide rate, which is twofold higher than the next highest national rate [2]. Certain occupations, such as farming and law enforcement, are associated with increased risk of firearm‐related suicide [3, 4, 5, 6, 7]. Previous studies identified occupational access to a firearm as increasing the odds of dying from firearm‐related suicide among working populations [8, 9], especially when the firearm was issued through work. However, these investigations suggest that other occupational‐related factors such as psychosocial stressors, including acute stress, may influence the relationship between occupational access to firearms and firearm‐related suicide [8, 10].

Job strain has been identified as a risk factor for suicide [3, 11, 12, 13, 14, 15, 16, 17]. While several models exist for examining job strain, one commonly used is Karasek's Job Demand‐Control Model, which was developed to predict how interactions between job demands and job control lead to mental strain at work [18]. Job demand is defined by stressors related to workload present in the work environment, whereas job control is defined by the ability to make decisions in the work environment [18]. According to Karasek's Job Demand‐Control Model, high job strain occurs when job demands are high and job control is low, whereas low job strain occurs when job demands are low and job control is high. This conceptualization of job strain has been widely used in occupational safety and health research for nearly 50 years [19, 20, 21, 22, 23, 24]. Job strain is associated with an increase in suicidal ideation [11, 12, 13, 14], suicide attempts [14, 25], and suicide [3, 15, 16]. It has also been associated with suicide among workers with occupational access to firearms as in farming [3, 17] and law enforcement [26, 27]. Reducing job strain through suicide prevention programming may reduce the risk of suicide and improve mental health of workers [28].

The purpose of this study was to examine the interaction of job strain as an effect modifier of the association between occupational access to firearms and firearm‐related suicide. We hypothesized that the magnitude of the association between occupational access to firearms and firearm‐related suicides would be higher among occupations with elevated levels of job strain, compared to those with lower levels of job strain. We also examined differences in lethal means used in suicides by sex [29, 30].

## 2 Materials and Methods

### 2.1 Study Design and Sample

We used a cross‐sectional study design to examine the effect of job strain on the association between occupational access to firearms and firearm‐related suicide. Suicide decedents were identified from the National Violent Death Reporting System (NVDRS), a case‐only, national population‐based surveillance system of all violence‐related fatalities (suicide, homicide) occurring in participating US states (see Appendix A for participating states, the District of Columbia, and Puerto Rico) [31, 32]. NVDRS data are abstracted from law enforcement reports, coroner/medical examiner reports, toxicology reports, and death certificates, and include characteristics of the suicide decedent (e.g., demographics, mental health, substance use, relationship and life stressors, job problems) and variables related to the weapon used (if any) [31]. Job strain was derived using data collected through the Occupational Information Network (O*NET), which is an online tool that provides information on occupational exposures, tasks, skills needed to perform the job, and abilities associated with the occupation (e.g., endurance, memorization, depth perception) by Standard Occupational Classification (SOC) code [33, 34]. O*NET has previously been used to examine how job exposures contribute to adverse health outcomes, such as mental health problems, occupational injuries, and suicide [33, 35]. SOC codes in O*NET were cross‐walked from 2019 O*NET‐SOC codes (eight‐digit codes specific to O*NET [36]) to the 2018 codes (six‐digit codes) recorded in the NVDRS using the NIOSH Industry and Occupation Computerized Coding System. The average occupational exposure scores of the eight‐digit SOC codes were calculated in cases where multiple such codes were associated with a single six‐digit SOC code. All O*NET data used were extracted from the 28.1 O*NET database version, the most recent release of the O*NET database [34]. Rural‐Urban Continuum Codes from 2023 were linked to counties of residence of the suicide decedents to evaluate rurality of residence as a confounding variable. Income data from the American Community Survey Integrated Public Use Microdata Series were linked to occupational groups to evaluate income as a confounding variable.

The SOC codes for suicide decedents recorded in NVDRS with an identifiable occupation at the time of death during 2013–2019 were examined. Suicide decedents categorized with an SOC major occupational group of “not in workforce” [97‐0000] (*n* = 19,266, 11.6%); “military” [98‐0000] (+*n* = 2892, 1.7%); or “missing, unknown, inadequate response to code” [SOC = 99‐0000] (*n* = 15,542, 9.3%) were excluded to ensure that the study population represented all noninstitutionalized civilians. We also excluded decedents who were younger than 25 years and older than 64 years. There was concern of underestimating suicides among young (< 25 years) and older (> 64 years) workers because young workers may likely have “student” entered as their occupation in the NVDRS, and older workers may likely have “retired” entered. In these cases, they would be coded in the NVDRS as “not in workforce” [SOC = 97‐0000] and therefore excluded from the study population [37]. Also, only suicide decedents with known lethal means were included, given that the analyses focused on suicide by firearm and non‐firearm, excluding 0.8% (*n* = 759) of the suicide decedents aged 25–64 years with an identifiable occupation at the time of death. Decedents with no known circumstances (i.e., any information available about the death) (*n* = 7834) were further excluded, as they may reflect missing information or poor quality data, as suggested by the NVDRS guidelines [31]. Following exclusions, 81,196 suicide decedents were included in our study sample after also excluding cases where the job strain measure could not be derived from the O*NET data.

### 2.2 Outcome

The outcome variable was firearm‐related suicides. Suicide was defined in NVDRS by ICD‐10 codes X60‐X84, Y87.0, and U03. Lethal means—defined in NVDRS as the type of weapon used to inflict the fatal injury [31]—was recorded in NVDRS as: firearm, non‐powder gun, sharp instrument, blunt instrument, poisoning, hanging/strangulation/suffocation, personal weapons (e.g., fists, feet, and hands for actions such as punching, kicking, and hitting), fall, explosive, drowning, fire/burns, motor vehicle (e.g., buses, motorcycles), other transport vehicle (e.g., trains, planes, boats), intentional neglect (e.g., starving oneself), biological weapons, shaking, other (e.g., taser, electrocution, nail gun, exposure to environment/weather), and unknown. We compared suicides where a firearm was used in the death to those where non‐firearm lethal means were used. Firearm‐related suicides consisted of all suicides that involved use of a firearm as recorded in the NVDRS. Non‐firearm‐related suicides consisted of all the other lethal means recorded in the NVDRS except for “unknown” and “missing.”

### 2.3 Exposure

The exposure variable was a dichotomous indicator for occupational groups where firearms are used in the course of the job [8, 38]. The occupation variable in NVDRS is defined as the usual occupation of the decedent as recorded on the death certificate which represents the longest held occupation by the decedent [31]. It is coded in NVDRS using the 2018 Standard Occupational Classification (SOC) code. Occupations were identified as having occupational access to firearms from the literature [8, 38] and are listed in Appendix B by major and broad SOC code (exposed group). Suicide decedents in occupational groups where firearms were not identified as used in the course of the job were included in the unexposed group.

### 2.4 Job Strain

Job strain was examined as a potential effect modifier and was operationalized using Karasek's Job Demand‐Control Model [18]. Previous studies [35, 39, 40, 41, 42] defined job demand using a mean of five O*NET variables (see Appendix C), and job control using the mean of eight O*NET variables (see Appendix D) [39]. The individual O*NET variables were each standardized by creating a Z score with the mean equal to 0 and standard deviation equal to 1 [39]. Measures of job demand and job control using O*NET variables were assessed for content validity and had high reliability (Cronbach's alpha = 0.66 for job demand; Cronbach's alpha = 0.85 for job control) [39, 41]. Job strain was defined as a four‐level qualitative variable using median cut points of the job demand measure and the job control measure to create the following categories: high strain job (high job demand, low job control), active job (high job demand, high job control), passive job (low job demand, low job control), and low strain job (low job demand, high job control) [39]. In this study, cut points for determining the categories of job strain were defined using the median values as described a priori from the literature, including the original research by Karasek et al. (1979) [18].

### 2.5 Statistical Analysis

Job strain levels were examined comparing suicide decedents with occupational access to firearms to decedents without occupational access to firearms. Differences in these distributions were assessed using $\chi^{2}$ tests. Dichotomous and continuous measures of job demand and job control were examined individually through an interaction term with occupational access to firearms. This interaction allowed us to determine whether the association between occupational access to firearms and firearm‐related suicide is moderated by job strain, which is a method used in prior studies [39, 40]. Percentages of job strain classifications for suicide decedents in the NVDRS data were also examined and compared to percentages of job strain classifications for population estimates in the American Community Survey Public Use Microdata Sample but were found to be similar and were not included in the modeling analyses.

Multivariable logistic regression was used to examine the effect of the interaction of job strain and occupational access to firearms on firearm‐related suicide, adjusting for decedent age, sex, education level, income, and rurality of residence. Unadjusted and adjusted model estimates were calculated. Unadjusted results are provided in Appendix E. Separate models were examined for biological sex, dichotomized into male and female suicide decedents, due to differences in lethal means used in suicides by sex [29, 30]. Additionally, job demand and job control measures were individually examined as effect modifiers on the association between occupational access to firearms and firearm‐related suicide, adjusting for the other job strain variable and for decedent age, sex, education level, income, and rurality of residence. All data analyses were conducted using SAS 9.4 (SAS Institute Inc, Cary, NC).

## 3 Results

**Figure 1:**
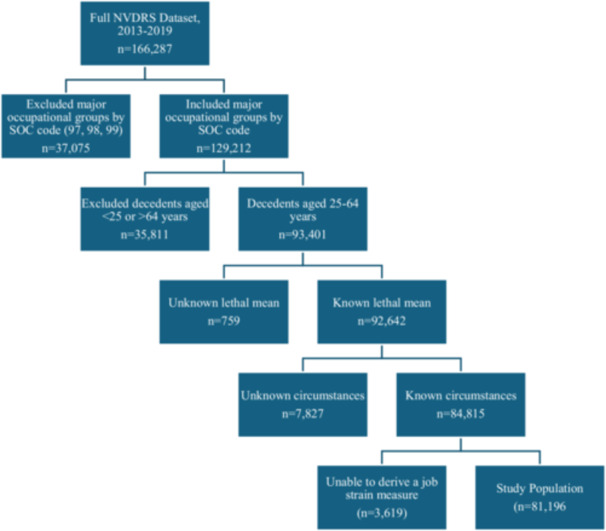
Flowchart of study participants. Data were drawn from the National Violent Death Reporting System (NVDRS), 2013–2019. Decedents were included if they were aged 25–64 years, had a known lethal means, and had documented circumstances of death. Only those whose Standard Occupational Classification (SOC) codes allowed derivation of the job strain measure were retained in the final sample (*n* = 81,196). NVDRS, National Violent Death Reporting System; SOC, Standard Occupational Classification.

After applying inclusion criteria, 81,196 suicide decedents were included in the analysis (Figure 1). Male and female decedents in the study population were most likely to be categorized as employed in a passive job (37.7% and 42.4%, respectively) (Table 1). Employment categorized as a low‐strain job (low demands‐high control) category had the lowest percentage among males (16.0%), whereas employment categorized as a high‐strain job (high demand‐low control) category had the lowest percentage among females (12.9%). Decedents with occupational access to firearms were most often employed in an active job compared to those without occupational access (males: 58.1% vs. 23.8%; females: 59.5% vs. 22.1%). About one‐quarter of male decedents with occupational access to firearms (25.1%) were employed in a low‐strain job compared to 15.7% of male decedents without occupational access to firearms. While a higher percentage of female decedents with occupational access to firearms were employed in high‐strain jobs compared to those without occupational access to firearms (22.7% and 12.8%, respectively), the opposite was observed for male decedents categorized as employed in high‐strain jobs with (15.7%) and without (21.5%) occupational access to firearms. Less than 2% of male and female decedents with occupational access to firearms were categorized as employed in a passive job (Table 1). After adjusting for confounding, decedents who had occupational access to firearms and were employed in a high strain job had a higher odds of dying from a firearm‐related suicide compared to those who did not have occupational access to firearms and were employed in a low‐strain job (Males: OR = 2.4, 95% CI: 1.9, 3.0, Table 2; Females: OR = 4.0, 95% CI: 2.4, 6.5, Table 3). Workers who had occupational access to firearms and worked in an active job had a higher odds of firearm‐related suicide for both male (OR = 3.0, 95% CI: 2.6, 3.4) (Table 2) and female (OR = 3.5, 95% CI: 2.6, 4.7) (Table 3) decedents, compared to those not having occupational access to firearms and being in a low‐strain job. Unadjusted results show similar findings (Appendix E).

**Table 1:** Frequency of suicide decedents with occupational access to firearms (*n* = 2691) and suicide decedents without access (*n* = 78,505), by job strain category and biological sex (Males: *n* = 64,437; Females: *n* = 16,759; *N* = 81,196) during 2013–2019.

Sex	Job Strain Level	Total Study Population		Suicide decedents with occupational access to firearms		Suicide decedents without occupational access to firearms		Chi‐square *p*‐value
		*n*	%	*n*	%	*n*	%	
Male	Low‐strain Job	10,335	16.0	599	25.1	9736	15.7	**< 0.001**
	Passive Job	24,268	37.7	26	1.1	24,242	39.1	
	Active Job	16,134	25.0	1387	58.1	14,747	23.8	
	High‐strain Job	13,700	21.3	375	15.7	13,325	21.5	
	**Total**	64,437	100.0	2387	100.0	62,050	100.0	
Female	Low‐strain Job	3663	21.9	53	17.4	3610	22.7	**< 0.001**
	Passive Job	7102	42.4	0	0	7102	43.2	
	Active Job	3823	22.8	181	59.5	3642	22.1	
	High‐strain Job	2170	12.9	69	22.7	2101	12.8	
	**Total**	16,759	100.0	303	100.0	16,456	100.0	

*Note:* Bolded *p*‐values: < 0.05.

**Table 2:** Adjusted odds ratios with 95% CIs examining the effect of job strain on the association between occupational access to firearms and firearm‐related suicide among decedents with occupational access to firearms (*n* = 2387) and workers without occupational access to firearms (*n* = 62,050) (males only) (*N* = 64,437) during 2013–2019.

Job Strain	Suicide Decedents with Occupational Access to Firearms		Suicide Decedents without Occupational Access to Firearms		Association between occupational access to firearms and firearm‐related suicide within strata of job strain^b^ OR^a^ (95% CI)
	*n*	OR^a^ (95% CI)	*n*	OR^a^ (95% CI)	
Low‐strain Job	599	**1.6 (1.3, 1.9)**	9736	Reference	**1.6 (1.3, 1.9)**
Passive Job	26	0.6 (0.3, 1.5)	24,242	1.0 (0.9, 1.0)	0.7 (0.3, 1.7)
Active Job	1387	**3.0 (2.6, 3.4)**	14,747	1.0 (1.0, 1.1)	**2.8 (2.4, 3.2)**
High‐strain Job	375	**2.4 (1.9, 3.0)**	13,325	**1.3 (1.2, 1.4)**	**1.8 (1.4, 2.3)**

*Note:* Bolded *p*‐values: < 0.05.

^a^ Adjusted for age, race/ethnicity, educational level, income, and rurality of residence.

^b^ Reference group includes suicide decedents without occupational access to firearms within each stratum.

**Table 3:** Adjusted odds ratios with 95% CIs examining the effect of job strain on the association between occupational access to firearms and firearm‐related suicide among decedents with occupational access to firearms (*n* = 304) and workers without occupational access to firearms (*n* = 16,455) (*N* = 16,759) (females only) (*N* = 16,759) during 2013–2019.

Job Strain	Suicide Decedents with Occupational Access to Firearms		Suicide Decedents without Occupational Access to Firearms		Association between occupational access to firearms and firearm‐related suicide within strata of job strain^b^ OR^a^ (95% CI)
	*n*	OR^a^ (95% CI)	*n*	OR^a^ (95% CI)	
Low‐strain Job	53	1.5 (0.9, 2.6)	3,610	Reference	1.5 (0.9, 2.6)
Passive Job	**‐‐**	**‐‐**	7,102	1.1 (1.0, 1.2)	**‐‐**
Active Job	181	**3.5 (2.6, 4.7)**	3,642	1.1 (0.9, 1.2)	**3.3 (2.3, 4.5)**
High‐strain Job	69	**4.0 (2.4, 6.5)**	2,101	**1.3 (1.1, 1.5)**	**2.8 (1.6, 4.8)**

*Note:* Bolded *p*‐values: < 0.05; ‐‐ Unknown values due to data suppression (< 10 decedents).

^a^ Adjusted for age, race/ethnicity, educational level, income, and rurality residence.

^b^ Reference group includes suicide decedents without occupational access to firearms within each stratum.

Examining job demand as a dichotomous variable in an interaction term and after adjusting for age, race/ethnicity, educational level, income, rurality of residence, and job control, decedents with occupational access to firearms in a high demand job had a higher odds of dying from firearm‐related suicide compared to decedents not having occupational access to firearms and in low demand jobs (Males: OR = 3.0, 95% CI: 2.7, 3.4; Females: OR = 3.5, 95% CI: 2.7, 4.6) (Table 4). Additionally, decedents with occupational access to firearms in low control jobs had a higher odds of dying from firearm‐related suicide compared to decedents not having occupational access to firearms and being in high control jobs (Males: OR = 1.8, 95% CI: 1.4, 2.2; Females: OR = 3.1, 95% CI: 1.9, 5.0) (Table 4).

**Table 4:** Adjusted odds ratios with 95% CIs examining the effect of dichotomous job demand and job control variables on the association between occupational access to firearms and firearm‐related suicide, comparing workers with occupational access to firearms (Males: *n* = 2387; Females: *n* = 304) and workers without occupational access to firearms (Males: *n* = 62,050; Females: *n* = 16,455) (Males: *N* = 64,437; Females: *N* = 16,759) during 2013–2019.

Sex	Level	Suicide Decedents with Occupational Access to Firearms		Suicide Decedents without Occupational Access to Firearms		Association between occupational access to firearms and firearm‐related suicide within strata of job demand levels^b^ ORs^a^ (95% CI)
		*n*	OR^a^ (95% CI)	*n*	OR^a^ (95% CI)	
Male	Low job demand	625	**1.6 (1.4, 1.9)**	33,978	Reference	**1.6 (1.4, 1.9)**
	High job demand	1762	**3.0 (2.7, 3.4)**	28,072	**1.2 (1.2, 1.2)**	**2.6 (2.4, 3.0)**
	Low job control	401	**1.8 (1.4, 2.2)**	37,567	Reference	**1.8 (1.4, 2.2)**
	High job control	1986	**2.1 (1.9, 2.3)**	24,483	**0.9 (0.8, 0.9)**	**2.3 (2.1, 2.6)**
Female	Low job demand	53	1.5 (0.9, 2.6)	10,712	Reference	1.5 (0.9, 2.6)
	High job demand	2282	**3.5 (2.7, 4.6)**	5743	**1.1 (1.0, 1.2)**	**3.3 (2.5, 4.3)**
	Low job control	2101	**3.1 (1.9, 5.0)**	9203	Reference	**3.1 (1.9, 5.0)**
	High job control	234	**2.4 (1.8, 3.1)**	7252	**0.9 (0.8, 1.0)**	**2.6 (2.0, 3.4)**

*Note:* Bolded *p*‐values: < 0.05.

^a^ Adjusted for age, race/ethnicity, educational level, income, rurality of residence, and either job control or job demand dependent on the tested interaction term.

^b^ Reference group includes suicide decedents without occupational access to firearms within each stratum.

## 4 Discussion

We observed job strain as an effect modifier on the association between occupational access to firearms and firearm‐related suicide. Workers with occupational access to firearms and employed in a high‐strain job had increased odds of firearm‐related suicide compared to those without occupational access to firearms and employed in a low‐strain job. This finding aligns with previous studies that suggest workers in high‐strain jobs are at higher risk for suicidal ideation [11, 12, 13, 14], suicide attempts [14, 25], and suicide [3, 15, 16]. Employment in a high‐strain job may lead to more impulsive decisions to die from suicide, especially when firearms are readily available in the workplace. Studies show that firearms in the home increase the risk of suicide because the firearm is easily accessible [43, 44, 45, 46, 47, 48]. We also observed that decedents with occupational access to firearms and working in an active job had a higher odds of firearm‐related suicide, compared to those without occupational access to firearms and working in a low‐strain job. These findings contrast with previous research on job strain and suicidality, which identified low job control as the primary contributor to suicide risk [25, 49, 50, 51]. Notably, two of these studies reported either no association between high job demands and suicidality [50] or even a reduced risk of suicidality in such contexts [51]. The discrepancy may stem from the present study's focus on firearm‐related suicides, particularly among individuals with occupational access to firearms. Prior research has indicated that acute stressors related to demands in one's personal life can elevate the risk of firearm‐related suicide [10]. While we were unable to determine whether the firearm was accessed at work or through the decedent's personal life, this finding aligns with prior research suggesting that greater familiarity with firearms overall may increase suicide risk [52, 53, 54]. Suicide prevention strategies should therefore consider how changes in job demands may influence the risk of firearm‐related suicide, especially in occupations where access to firearms is a routine part of the job.

Both male and female decedents who had occupational access to firearms and worked in either high‐strain jobs or active jobs had higher odds of firearm‐related suicide; however, the odds were higher among female decedents. This may be due, in part, to the comparison group—females without occupational access to firearms or females in general—having less personal access to firearms compared to their male counterpoints [9]. The only difference between sexes was that female decedents who work in low‐strain jobs did not have an increased odds of firearm‐related suicide when they have occupational access to firearms. This may suggest that female workers are at particularly elevated risk for firearm‐related suicide in high stress environments.

More recently, the Job Demand‐Control Model, which was used in this study, has been modified since its conception to include social support as an additional metric of job strain [55]. A measure of workplace social support was not derived for this study because a validated measure for this metric using O*NET variables does not exist [34, 35, 39, 40, 41, 42]. Having a strong social support network at work has been shown to reduce job strain [56], which may affect the association between occupational access to firearms and firearm‐related suicide. In fact, occupational groups with occupational access to firearms including police officers [57, 58, 59] and farmers [60, 61, 62] report low social support in the workplace, and therefore the impact of social support is important to understand in context with job demands and job control.

Interventions aimed at reducing firearm‐related suicide should address job strain, particularly among workers with occupational access to firearms. Various strategies already exist to mitigate job strain, especially in professions like law enforcement [63, 64, 65, 66]. However, these interventions should also consider the role of occupational access to firearms and other lethal means. While police organizations often caution against the direct removal of issued firearms [26, 27]—which may make addressing job strain a more feasible option— voluntary and temporary firearm storage during periods of high stress may be both acceptable and effective in reducing suicide risk. Evidence from a prior study indicates that law enforcement officers may be more willing to adopt procedures that reduce firearm access during periods of elevated risk after receiving suicide‐prevention training [67]. Research supports the effectiveness of such voluntary storage in lowering the likelihood of firearm‐related suicide in military populations [26, 68]. Additionally, interventions should incorporate discussions around safe storage practices for firearms and other lethal means. Programs like Conversations on Access to Lethal Means (CALM) have shown to be effective in engaging lay audiences on this topic [69] and could be adapted for workers with occupational firearm access.

This study has several strengths. First, this study used NVDRS data, which include decedents from nearly all US states, the District of Columbia, and Puerto Rico, which suggests results are generalizable to US workers. Second, this study was one of the first to examine how job strain affects the association between occupational access to firearms and firearm‐related suicide among US workers. Finally, we provide robust evidence that job strain is an important characteristic to consider in assessing occupational suicide risk. Furthermore, we examined the job demand and job control measures as dichotomous and continuous variables and found the effect modification results to be similar to the job strain measure when median cut points of the job demand and job control measures were used, suggesting the results are robust to different modes of classification [39].

This study also has some limitations. First, there may be exposure misclassification, as not all workers within the included occupational groups with occupational access to firearms would have direct access to firearms in their respective jobs. We also may have non‐differentially misclassified workers in occupational groups without access to firearms when they may have direct access in their respective job. However, we attempted to be conservative with this categorization by using previous literature [8, 38]. Second, there may be misclassification in how decedents were categorized by occupation or by the lethal means used; however, both approaches align with recommended NVDRS classification procedures [31]. Third, we were unable to adjust for personal firearm ownership or other access to firearms (i.e., firearm ownership of a family member), which has been suggested to increase the risk of firearm suicide coupled with having acute stress [10]. However, findings from this study assist in understanding how having access to and use of firearms at work is associated with firearm‐related suicide compared to workers in occupations without occupational access to firearms. Fourth, dead controls were used as the comparison group for the outcome, which may introduce selection bias [70]. However, we compared rates of non‐firearm‐related suicide between exposure groups and found that they had similar rates, suggesting selection bias does not explain our observed results.

## 5 Conclusions

Occupational access to firearms and working in a high‐strain job or active job contributed to greater odds of firearm‐related suicide in both male and female decedents, compared to those without occupational access to firearms and working in a low‐strain job. Findings from this study provide some of the first epidemiological evidence for how job factors like job strain contribute to firearm‐related suicide among US workers.

## Author Contributions

Victor A. Soupene is the principal investigator of the study, and was involved with drafting the manuscript, conducting the statistical analysis, and is responsible for the overall study and final decisions on the analysis and manuscript. Jonathan Davis was involved with conducting and revising the statistical analysis and manuscript. Jonathan M. Platt, Paul A. Romitti, and Joseph E. Cavanaugh were involved in revising the analysis and manuscript. Carri Casteel was responsible for overseeing the study and was involved in conducting and revising the statistical analysis and manuscript.

## Ethics Statement

This study was not considered human subjects research by the University of Iowa Human Subjects Office (IRB # 202401135).

## Data Availability

Data used in this study are from the National Violent Death Reporting System (NVDRS) Restricted Access Database (RAD), managed by the Centers for Disease Control and Prevention (CDC). Access to the NVDRS RAD is restricted and requires approval from the CDC.

## Appendix A

**Table A1:** States contributing to the NVDRS by year, 2013–2019.

State	2013	2014	2015	2016	2017	2018	2019
Alabama					c	X	X
Alaska	X	X	X	X	X	X	X
Arizona		c	X	X	X	X	X
Arkansas						c	X
California				c	X^a^	X^a^	X^a^
Colorado	X	X	X	X	X	X	X
Connecticut		c	X	X	X	X	X
Delaware				c	X	X	X
Washington DC				c	X	X	X
Florida							c
Georgia	X	X	X	X	X	X	X
Hawaii		c	X	X	b	b	X
Idaho							c
Illinois			c	X^a^	X^a^	X^a^	X^a^
Indiana			c	X	X	X	X
Iowa			c	X	X	X	X
Kansas		c	X	X	X	X	X
Kentucky	X	X	X	X	X	X	X
Louisiana					c	X	X
Maine		c	X	X	X	X	X
Maryland	X	X	X	X	X	X	X
Massachusetts	X	X	X	X	X	X	X
Michigan		X	X	X	X	X	X
Minnesota		c	X	X	X	X	X
Mississippi							c
Missouri					c	X	X
Montana						c	X
Nebraska					c	X	X
Nevada				c	X	X	X
New Hampshire		c	X	X	X	X	X
New Jersey	X	X	X	X	X	X	X
New Mexico	X	X	X	X	X	X	X
New York		c	X	X	X	b	X
North Carolina	X	X	X	X	X	X	X
North Dakota						c	X
Ohio	X	X	X	X	X	X	X
Oklahoma	X	X	X	X	X	X	X
Oregon	X	X	X	X	X	X	X
Pennsylvania			c	X^a^	X^a^	X^a^	X^a^
Rhode Island				c	X	X	X
South Carolina	X	X	X	X	X	X	X
South Dakota							c
Tennessee							c
Texas						c	b
Utah	X	X	X	X	X	X	X
Vermont		c	X	X	X	X	X
Virginia	X	X	X	X	X	X	X
Washington			c	X^a^	X^a^	X	X
West Virginia				c	X	X	X
Wisconsin	X	X	X	X	X	X	X
Wyoming						c	X

*Note:* X = Reported data for violent deaths from the entire state; X^a^ = Reported data for violent deaths that occurred among selected counties within the state but did not include violent deaths for all counties within the state.

^b^ = Excluded by Centers for Disease Control and Prevention from the NVDRS due to incomplete data reporting.

^c^ = Year the state enrolled in the NVDRS.

See Table A1.

## Appendix B

**Table B1:** Occupations (by major and broad SOC code) with occupational access to firearms and job strain level.

Major SOC code	Occupation (with broad SOC code)	Job Strain Level
Management Occupations (11‐0000)	Farmers, Ranchers, and Other Agricultural Managers (11‐9010)	Low‐strain Job
Protective Services Occupations (33‐0000)	First‐Line Supervisors of Law Enforcement Workers (33‐1010)	Active Job
	Bailiffs, Correctional Officers, and Jailers (33‐3010)	High‐strain Job
	Detectives and Criminal Investigators (33‐3020)	Active Job
	Fish and Game Wardens (33‐3030)	Active Job
	Police Officers (33‐3050)	Active Job
	Animal Control Workers (33‐9010)	Active Job
	Private Detectives and Investigators (33‐9020)	Active Job
	Security Guards and Gaming Surveillance Officers (33‐9030)	Active Job
Farming, Fishing, and Forestry Occupations (45‐0000)	First‐Line Supervisors of Farming, Fishing, and Forestry Workers (45‐1010)	Low‐strain Job
	Fishing and Hunting Workers (45‐3030)	Passive Job
	Forest and Conservation Workers (45‐4010)	Passive Job

See Table B1.

## Appendix C

**Table C1:** Job demand variables from O*Net.

Variable Name	O*Net Construct	O*Net Description	O*Net Question
Selective attention	Abilities	Ability to concentrate on a task over a period of time	How important is selective attention to the performance of your current job? (Scale: Not Important = 1 – Extremely Important = 5)
Attention to details	Work styles	Work requires being careful with detail for work tasks	How important is attention to detail to the performance of your current job? (Scale: Not Important = 1 – Extremely Important = 5)
Importance to being exact	Work context	Importance of being exact or accurate in performing the job	How important to your current job is being very exact or highly accurate? (Scale: Not Important = 1 – Extremely Important = 5)
Consequences of error	Work context	Seriousness of a result if a worker made a mistake that is not readily correctable	How serious a mistake can you make on your current job (one you can't easily correct)? (Scale: Not serious at all = 1 – Extremely serious = 5)
Time sharing	Abilities	Ability to shift back and forth between multiple tasks	How important is time sharing to the performance of your current job? (Scale: Not Important = 1 – Extremely Important = 5)

See Table C1.

## Appendix D

**Table D1:** Job control variables from O*Net.

Variable Name	O*Net Construct	O*Net Description	O*Net Measurement
Achievement/Effort	Work styles	Work requires challenging achievement goals and effort to mastering work tasks	How important is achievement/effort to the performance of your current job? (Scale: Not Important = 1 ‐ Extremely Important = 5)
Active learning	Basic skills	Recognizing implications of novel information for current and future decision‐making tasks	How important is active learning to the performance of your current job? (Scale: Not Important = 1 ‐ Extremely Important = 5)
Thinking creatively	Work activities	Developing or creating new ideas, applications, systems, or products with artistic contributions	How important is thinking creatively to the performance of your current job? (Scale: Not Important = 1 ‐ Extremely Important = 5)
Independence	Work styles	Job requires the worker to do things on their own with little to no supervision	How important is independence to the performance of your current job? (Scale: Not Important = 1‐Extremely Important = 5)
Impact of decision on work	Work context	Results of the decision impact others or the image of the employer	In your current job, what results do your decisions usually have on other people or the image or reputation or financial resources of your employer? (Scale: No results = 1 – Very important results = 5)
Frequency of decision making	Work context	Frequency in the work making decisions that affects others or the employer	In your current job, how often do your decisions affect other people or the image or reputation or financial resources of your employer? (Scale: Never = 1 ‐ Every day = 5)
Freedom to make decisions	Work context	Freedom to make decisions without supervision	In your current job, how much freedom do you have to make decisions without supervision? (Scale: No freedom = 1 – A lot of freedom = 5)
Structured work	Work context	Job structured as opposed to the worker determining tasks, priorities, and goals	How much freedom do you have to determine the tasks, priorities, or goals of your current job? (Scale: No freedom = 1 – A lot of freedom = 5)

See Table D1.

## Appendix E

**Table E1:** Unadjusted odds ratios with 95% CIs examining the effect of job strain on the association between occupational access to firearms and firearm‐related suicide among workers with occupational access to firearms (Males: *n* = 2387; Females: *n* = 304) and workers without occupational access to firearms (Males: *n* = 62,050; Females: *n* = 16,455) by sex (Males: *N* = 64,437; Females: *N* = 16,759).

Sex	Variable	Suicide Decedents with Occupational Access to Firearms		Suicide Decedents without Occupational Access to Firearms	
		*n*	OR (95% CI)	*n*	OR (95% CI)
Male	Low job strain	599	**1.8 (1.5, 2.1)**	9736	1.0
	High job strain	375	**2.5 (2.0, 3.2)**	13,325	**1.2 (1.1, 1.3)**
Female	Low job strain	53	**1.8 (1.1, 3.0)**	3610	1.0
	High job strain	69	**3.9 (2.5, 6.3)**	2101	1.1 (1.0, 1.2)
Male	Low job strain	599	**1.8 (1.5, 2.1)**	9736	1.0
	Active job	1387	**2.7 (2.4, 3.1)**	14,747	**1.1 (1.0, 1.1)**
Female	Low job strain	53	**1.8 (1.1, 3.0)**	3610	1.0
	Active job	181	**3.2 (2.4, 4.2)**	3642	1.1 (1.0, 1.2)
Male	Low job strain	599	**1.8 (1.5, 2.1)**	9736	1.0
	Passive job	26	0.5 (0.2, 1.0)	24,242	**0.8 (0.8, 0.8)**
Female	Low job strain	53	**1.8 (1.1, 3.0)**	3610	1.0
	Passive job	‐‐	NC	7102	0.9 (0.9, 1.0)

*Note:* Bolded *p*‐values: < 0.05; ‐‐Unknown values due to data suppression (< 10 decedents). Reference group includes suicide decedents without occupational access to firearms within each stratum.

See Table E1.
